# m^6^A Regulator-Based Methylation Modification Patterns Characterized by Distinct Tumor Microenvironment Immune Profiles in Rectal Cancer

**DOI:** 10.3389/fonc.2022.879405

**Published:** 2022-07-06

**Authors:** Kaili Liao, Jialing Hu, Yu Huang, Siji Yu, Qijun Yang, Fan Sun, Chengfeng Wu, Yunqi Cheng, Wenyige Zhang, Xue Zhang, Hongyu Li, Xiaozhong Wang

**Affiliations:** ^1^ Jiangxi Province Key Laboratory of Laboratory Medicine, Jiangxi Provincial Clinical Research Center for Laboratory Medicine, Department of Clinical Laboratory, The Second Affiliated Hospital of Nanchang University, Nanchang, China; ^2^ Department of Emergency medicine, The Second Affiliated Hospital of Nanchang University, Nanchang, China; ^3^ School of Advanced Manufacturing of Nanchang University, Nanchang, China; ^4^ Queen Mary College of Nanchang University, Xuefu Road, Nanchang, Nanchang, China; ^5^ Department of Vascular Surgery, The Second Affiliated Hospital of Nanchang University, Nanchang, China

**Keywords:** rectal cancer, M6A modification, tumor microenvironment, immune profiles, immunotherapy

## Abstract

**Background:**

Previous studies reported the related role of RNA n6-methyladenosine (m6A) modification in tumorigenesis and development. However, it is not clear whether m6A modification also plays a potential role in the immune regulation of rectal cancer (RC) and the formation of tumor microenvironment.

**Methods:**

In this study, we screened 23 m6A regulatory factors from 369 rectal cancer specimens, further determined the modification patterns of m6A in RC, and systematically linked these modification patterns with the characteristics of TME cell infiltration. The principal component analysis (PCA) algorithm was used to evaluate the m6A modification pattern of a single tumor related to immune response.

**Results:**

Three different m6A modification patterns were found in the measurement results, which are related to different clinical results and biological pathways. TME identification results show that the identified m6A pattern is closely related to immune characteristics. According to the m6Ascore extracted from m6A-related signature genes, RC patients were divided into high and low score subgroups combined with tumor mutation burden. Patients with high tumor mutation burden and higher m6Ascore have a significant survival advantage and enhanced immune infiltration. Further analysis showed that patients with higher m6Ascore had higher PD-L1 expression levels and showed better immune response and lasting clinical benefits.

**Conclusions:**

M6A modification plays a crucial role in the formation of TME diversity and complexity. The evaluation of the m6A modification mode will help us to enhance our understanding of the characteristics of TME infiltration and provide new insights for more effective immunotherapy strategies.

## Introduction

Rectal cancer is a malignant tumor that occurs in the lower part of the large intestine, accounting for 30-40% of colorectal cancer (CRC) (1, 2). There are approximately 700,000 confirmed cases of rectal cancer each year in the world, and the annual death toll is approximately 310,000 (3). Although the colon and rectum are anatomically related, there are significant differences in recurrence rates and treatment options between cancer types (4). Therefore, the determination of reliable prognostic biomarkers to improve the prognosis of rectal cancer has important clinical biological significance. The molecular mechanism of the occurrence and development of malignant tumors is one of the current research hotspots. RC is a major social health problem and occupies a special position in tumor diseases. The interaction network jointly established by immune cells, endothelial cells, mesenchymal fibroblasts and matrix-related molecules in the tumor and surrounding tissues constitutes the TME (5). Tumor cells in TME can directly or indirectly invade tissues through blood vessels and lymphatic vessels, and infiltrating cells can induce immune responses by releasing cytokines, cytokine receptors and other factors, and affect tumor progression (6–9). However, the role of TME and the specific biological mechanism of potential therapeutic response are still unclear (10).

In recent years, the study of post-transcriptional gene regulation in eukaryotes has opened up new fields. So far, more than 100 different chemical modifications have been discovered for post-transcriptional modification (11). The methylation of messenger RNA to form m6A is considered to be the most abundant internal modification in messenger RNA and has become a wide-ranging regulatory mechanism that controls gene expression in various physiological processes (12–16). m6A modification is a dynamic and reversible process in mammalian cells. It is installed by m6A methyltransferases, removed by m6A demethylases, and recognized by reader proteins. The process is regulated by methyltransferase, demethylase and binding protein, also known as “writer”, “erasers” and “reader” (17, 18). Post-transcriptional modification has become an important regulator of various physiological processes and disease progression, and has attracted increasing attention in biological science research. In addition, in terms of molecular mechanism, m6A participates in almost all steps of RNA metabolism, including translation, degradation, splicing, export and folding of mRNA (19, 20).

Recent literature has reported the interaction between TME infiltration of immune cells and m6A modification, which cannot be fully explained by the mechanism of RNA degradation. Studies have shown that m6A methylation of dendritic cells and YTHDF1 regulate anti-tumor immunity, further supporting the view that decreased YTHDF1 expression may be related to T cell inflammation and tumor microenvironment (21). Another study showed that m6A mRNA demethylase FTO regulates the tumorigenicity of melanoma and the response to PD-1 blockade, and plays an important role in the response to immunotherapy (22). In addition, it is reported that Mettl3-mediated m6A modification plays an important role in promoting the maturation and activation of dendritic cells, which may promote cancer immunotherapy (23). However, the above research is limited to one or two m6A modulators and cell types, and the anti-tumor effect requires the interaction of multiple tumor suppressor factors. Therefore, the systematic evaluation of the infiltration characteristics of TME cells mediated by multiple m6A regulatory factors will comprehensively strengthen our understanding of TME immune regulation.

In this study, we screened the genomic information of 369 rectal cancer samples from The Cancer Genome Atlas (TCGA) (167) and Gene-Expression Omnibus (GEO) databases (203), systematically evaluated m6A modification patterns, and deeply understood the potential connection between m6A modification patterns and TME cell infiltration characteristics. Three different m6A modification modes are summarized. What is interesting is that the TME characteristics of these three modes are highly consistent with the immune exclusion phenotype, the immune inflammation phenotype and the immune desert phenotype, respectively. This indicates that the role of m6A modification in shaping individual tumor microenvironmental characteristics is a promising method. To this end, we established a scoring system to quantify the m6A modification patterns of individual patients.

## Materials and Methods

### Collect and Organize of Expression Datasets Obtained From Public Databases

With the rapid development of precision medicine, researchers are increasingly using statistical algorithms to explore new diagnosis and treatment goals. We retrospectively collected gene expression data and related data of RC samples from the GEO database (https://www.ncbi.nlm.nih.gov/geo/), TCGA (https://cancergenome.nih.gov/) public data sets and clinical characteristics data. In short, the study collected TCGA and GSE87211 cohorts for further analysis. Through Protein-Protein Interaction (PPI) network analysis, the key nodes in the differentially expressed proteins were found and visualized by Cytoscape.

### Consensus Molecular Cluster Analysis of Twenty-Three m6A Regulators

In the process of cluster analysis, the cohort with fewer m6A regulatory factors was not included. Twenty-three regulatory factors were extracted from the TCGA and GSE87211 cohorts to identify different m6A modification patterns mediated by m6A regulatory factors. 23 m6A regulators including 8 writers (METTL3, METL14, METL16, WTAP, VIRMA, ZC3H13, RBM15 and RBM15B), 2 erasers (FTOA and ALKBH5), 13 readers (YTHDC1, YTHDC2, YTHDF1, YTHDF2, YTHDF3, HNRNPC, FMR1, LRPPRC, HNRNPA2B1, IGFBP1, IGFBP2, IGFBP3 and RBMX). Subsequently, we applied an unsupervised cluster analysis method to determine different m6A modification patterns based on the expression of 23 m6A regulatory factors, and classified patients for further analysis. The number and stability of clustering are determined by the consensus clustering algorithm. We use the Consensus Cluster Plus package to repeat the above steps to ensure the stability of the classification results.

### Gene Set Variation Analysis (GSVA) and Gene Ontology (GO) Kyoto Encyclopedia of Genes and Genomes (KEGG) Annotation

In order to study the biological process differences between the m6A modification modes, we used the “GSVA” R package to perform GSVA enrichment analysis. GSVA is a non-parametric, unsupervised method, usually used to estimate changes in pathways and biological process activity in expression data sets (24). The adjusted P value <0.05 indicates that the difference is statistically significant. We used cluster Profiler R package to annotate m6A-related genes with a cutoff value of FDR <0.05. And ggplot was used to visualize the enrichment analysis.

### Estimation of m6A Modification Pattern in Immune Cell Infiltration

We used single sample gene set enrichment analysis (ssGSEA) to evaluate the relative abundance of each cell infiltration in RC TME. According to published research methods, the gene set of each TME infiltrating immune cell type is labeled (25, 26). Immune cell subtypes include activated CD4 T cells, activated B cells, mast cells, monocytes, etc (25). The enrichment score calculated by ssGSEA analysis represents the relative abundance of each TME infiltrated cell in each sample.

### Screening of m6A Differentially Expressed Genes (DEGs)

In order to identify m6A-related genes, we divided patients into three different m6A modification modes based on the expression of 23 m6A regulatory factors. The empirical Bayesian method was used to quantitatively analyze the DEGs between different modification modes (27). The significance standard for determining DEG is the corrected P value <0.001.

### Construction of the m6Ascore

We constructed a scoring system to evaluate the m6A modification pattern of individual patients with rectal cancer-m6A gene characteristics to quantify the m6A modification pattern of individual tumors, and named it m6Ascore. In simple terms, first normalize DEGs from different m6Aclustered samples in the sample, using unsupervised clustering method to analyze the extracted overlapping DEGs, then use consensus clustering algorithm to determine the number and stability of gene clusters, and use univariate Cox. The regression model performs prognostic analysis on each gene in the signature. Genes with significant prognosis were extracted for subsequent analysis. PCA analysis is performed on the final gene expression profile, and principal component 1 and principal component 2 are selected as the signature scores. The advantage of this method is that the score is concentrated on the set with the largest correlation (or irrelevant) gene block in the set, and the weight of the gene contribution that is not tracked with other set members is reduced. Then, we use a method similar to the previous study to define the m6Ascore (28, 29): m6Sig score=∑(PC1i+PC2i), where is the final expression of the m6A phenotype-related genes.

### Statistical Analysis

For quantitative data, the statistical significance of normally distributed variables was estimated by Student’s t test, and the Wilcoxon rank sum test was used for non-normally distributed variables. For the comparison of more than two groups, the nonparametric method uses the Kruskal-Wallis test, the parametric method uses the one-way analysis of variance. The Kaplan-Meier survival analysis and the Cox proportional hazard model, and the R package “Survminer” are used to analyze the relationship between the m6A modification model and the prognosis Relationship. The measurement cut function in the “survival” software package was used to stratify the samples into high m6Sig score subgroups and low m6Ascore subgroups. P <0.05 is considered statistically significant.

## Results

### Genetic Variation of m6A Regulators in Rectal Cancer

**Figure 1:**
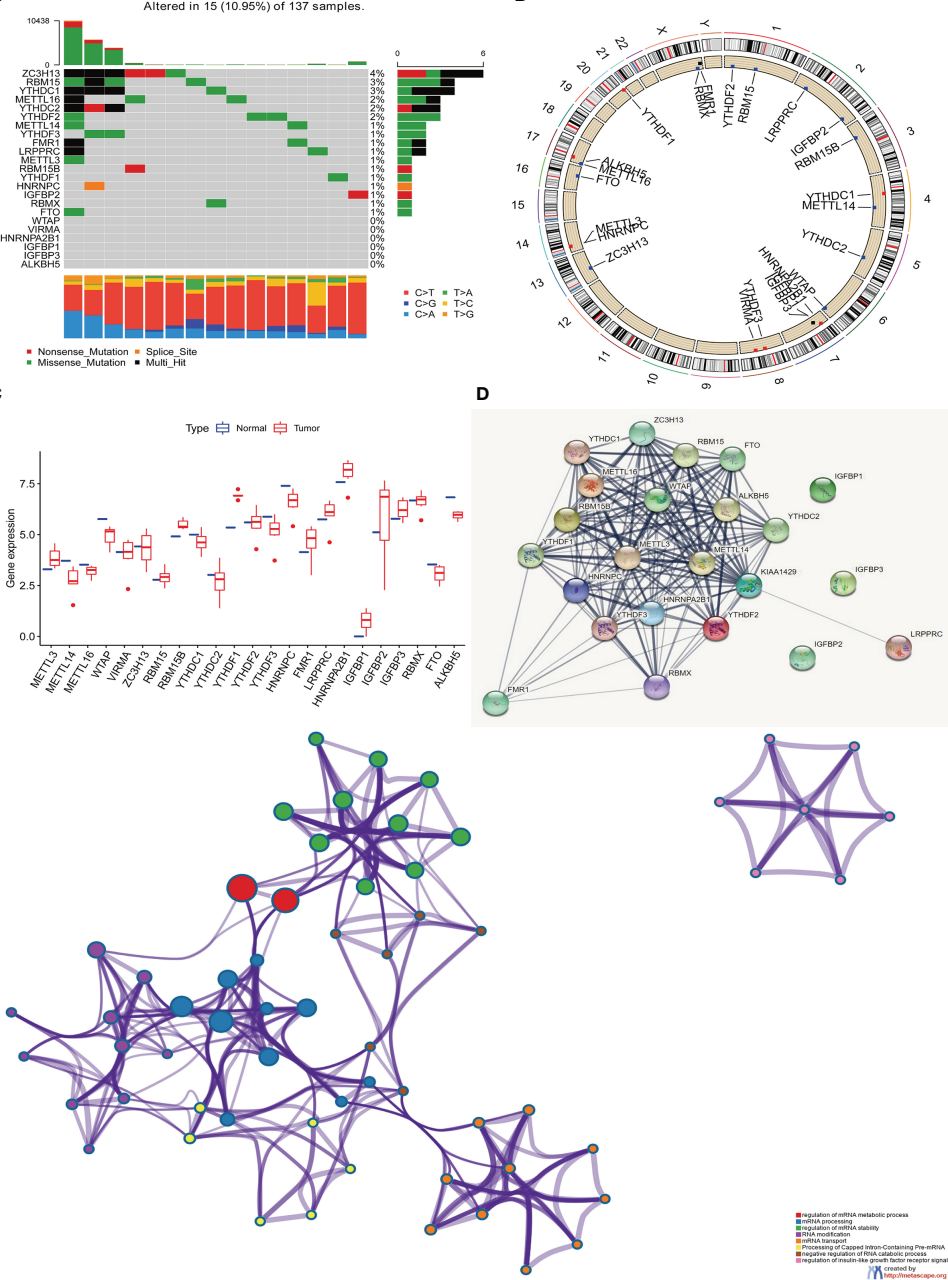
The genetic and expression variant landscape of m^6^A regulators in rectal cancer. **(A)** The mutation frequency of 23 m^6^A regulatory factors in 137 rectal cancer patients in the TCGA-RC cohort. Each column represents an .individual patient. The bar graph above shows TMB, and the numbers on the right indicate the mutation frequency of each regulatory factor. The bar graph on the right shows the proportion of each variant type and the four different colors indicates four variant types with legends showing in the bottom of the figure. The stacked bar graph below shows the proportion of different single nucleotide mutation in each sample, with annotation on the right showing six colors representing six different mutations. **(B)** Use the GSE87211 cohort to locate the changes of m^6^A regulatory factor CNV on 23 chromosomes. **(C)** The expression of 23 m6A regulatory factors in normal tissues and rectal tissues. Tumor, red; normal, blue. The upper and lower ends of the box represent a quarter of the value range. The line in the box represents the median value, and the black dots represent the outliers. **(D)** The protein protein interaction network(PPI) demonstrated the interaction between 23 m^6^A regulatory factors, including 23 nodes and 104edges. **(E)** The enrichment analysis of 23 m^6^A regulatory factors. These m^6^A regulatory factors have a rich regulatory role in the metabolism of mRNA, primarily involved in the mRNA metabolic process, mRNA processing and mRNA stability.

In this study, we studied the role of 23 m6A RNA methylation regulation genes in RC (“writer”: METTL3, METTL14, METTL16, WTAP, VIRMA, ZC3H13, RBM15 and RBM15B; “reader”: YTHDC1 YTHDC2, YTHDF1, YTHDF2, YTHDF3, HNRNPC, FMR1, LRPPRC, HNRNPA2B1, IGFBP1, IGFBP2, IGFBP3 and RBMX; there are also “ erasers”: FTO and ALKBH5). We first determined the incidence of somatic mutations in 23 m6A regulatory factors in RC. 15 of 137 samples (10.95%) experienced genetic changes in m6A regulatory factors, including missense mutations, nonsense mutations, and Multi -Hit and Splice-site. Among them, ZC3H13 has the highest mutation frequency, followed by RBM15 and YTHDC1. WTAP, ZC3H13, VIRMA, HNRNPA2B1, IGFBP1, IGFBP3 and ALKBH5 have no mutations (**Figure 1A**). Next, we conducted an in-depth analysis of the position of the copy variation (CNV) of the 23 m6A regulatory factors on the chromosome, as shown in (**Figure 1B**). In order to determine whether genetic variation affects the expression of m6A regulatory factors in RC patients, we studied the expression levels of regulatory factors in normal and RC samples and found that, compared with normal control samples, METTL14, METTL16, WTAP, YTHDC1, YTHDC2, YTHDF3, HNRNPC, FTO, and ALKBH5 were significantly down-regulated in tumor samples, while METTL13, RBM15, RBM15B, FMR1, LRPPRC, HNRNPA2B1, IGFBP1, IGFBP2, and IGFBP3 were significantly up-regulated in tumor samples (**Figure 1C**). In order to further study the role of 23 m6A regulatory factors in RC, we created PPI and visualized it with Cytoscape software, including 23 nodes and 104 edges (**Figure 1D**). These regulatory factors are abundant in the process of regulation of mRNA metabolism process and mRNA stability, RNA modification and mRNA transport are many important ways (**Figure 1E**).

The above analysis shows that there are significant differences and connections in the genome and transcriptome landscape of m6A regulatory factors between normal and RC samples. Therefore, the expression changes and genetic variation of m6A regulatory factors play an important role in regulating the occurrence and development of RC. In view of the relatively high mutation frequency of the author’s gene ZC3H13, we analyzed the expression differences of 23 m6A regulatory factors in ZC3H13 wild-type (**Supplementary Figures 1A–W**).

### The Relationship Between the High and Low Expression Groups of 23 m6A Regulators and the Overall Survival of Rectal Cancer

**Figure 2:**
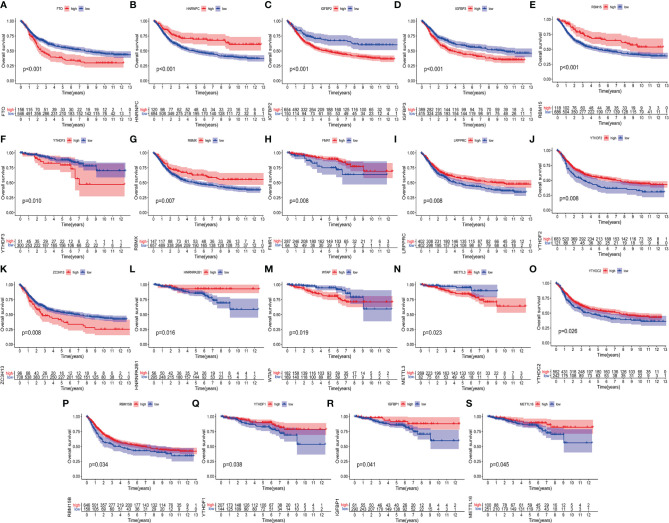
Kaplan-Meier curves of 19 m^6^A regulatory factors in patients with rectal cancer. **(A–S)** Each figure represents the comparison of the overall survival in patients with higher and low expression of a specific gene and the gene name is shown in the top of each figure. The ordinate shows the survival probability and the abscissa shows the years of survival of the patients. The number of patients in high- and low-expression groups are shown in the bottom of each figure. Log-rank test P<0.05 indicates that the difference is statistically significant.

On the TCGA and GSE87211 data sets with OS data and clinical information, the Kaplan-Meier method was used to analyze the prognosis of the survival curves of 23 m6A regulatory factors. The results showed that the expression of 19 m6A regulatory factors was related to prognosis. In short, compared with the low expression group of corresponding regulatory factors, HNRNPC, RBM15, RBMX, FMR1, LRPPRC, YTHDF2, HNRNPA2B1, YTDC2, RBM15B, YTHDF1, IGFBP1 and The METTL16 high expression group showed a significant survival advantage. In contrast, FTO, IGFBP2, IGFBP3, YTHDF3, ZC3H13, WTAP and METTL13 showed significant survival advantages in their corresponding low expression groups **(** **Figures 2A–S** and **Table 1** **)**.

**Table 1:** Analysis of 23 m6A regulatory factors through univariate Cox regression.

id	HR	HR.95L	HR.95H	p-value
METTL3	1.3705	0.6186	3.0366	0.4374
METTL14	0.8918	0.4358	1.8253	0.7541
METTL16	0.4158	0.1555	1.1119	0.0804
WTAP	2.7722	0.9954	7.7205	0.0510
VIRMA	0.7447	0.3319	1.6713	0.4748
ZC3H13	1.0227	0.6227	1.6798	0.9292
RBM15	0.9465	0.4822	1.8579	0.8730
RBM15B	0.9631	0.4989	1.8593	0.9109
YTHDC1	1.0778	0.7387	1.5726	0.6975
YTHDC2	1.1251	0.6651	1.9033	0.6602
YTHDF1	0.7173	0.3680	1.3983	0.3293
YTHDF2	1.1000	0.3771	3.2083	0.8615
YTHDF3	0.9860	0.4498	2.1614	0.9719
HNRNPC	1.0862	0.3652	3.2308	0.8818
FMR1	0.4990	0.2426	1.0262	0.0588
LRPPRC	0.6365	0.3369	1.2028	0.1641
HNRNPA2B1	0.6183	0.2274	1.6808	0.3460
IGFBP1	0.9685	0.8126	1.1544	0.7210
IGFBP2	1.0951	0.8981	1.3353	0.3691
IGFBP3	1.2071	0.8644	1.6858	0.2693
RBMX	0.8887	0.3850	2.0514	0.7822
FTO	1.2640	0.8290	1.9274	0.2763
ALKBH5	1.4732	0.5645	3.8446	0.4285

### m6A Methylation Modification Patterns Mediated by 23 Regulators

**Figure 3:**
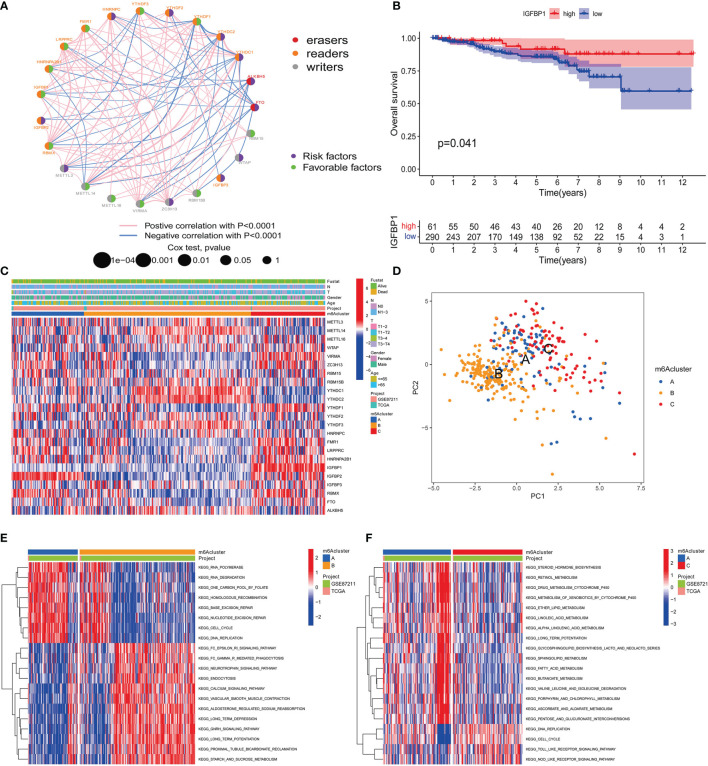
m6A methylation modification patterns and related clinical features. **(A)** The interaction of m^6^A regulatory factors in rectal cancer. The size of the circle represents the influence of each adjusting factor on the prognosis, and the range of the calculated value of the Log-rank test is p<0.0001, p<0.001, p<0.01, p<0.05, p<1. The purple dots in the circle indicate prognostic risk factors; the green dots in the circle indicate prognostic protective factors. The line connecting the regulators represents the interaction between them, and the thickness represents the relative strength between the regulators. Blue is a negative correlation, and pink is a positive correlation. The regulatory factors (“eraser”, “reader”, and “writer”) are marked in red, brown, and gray, respectively. **(B)** Kaplan-Meier curve of the patient group with high and low expression of IGFBP1. The number of high and low expression groups of patients were indicated in the bottom. Log-rank test, P = 0.041. **(C)** Unsupervised aggregation of 23 m^6^A regulatory factors in the GSE87211 rectal cancer cohort. Survival status, tumor stage, gender, age, project and m^6^Acluster are used as patient annotations. On the right, the bar with two colors indicates the level of gene expression and red color represents high expression while blue represents low expression. m6A cluster A–C were shown in blue, yellow and red, respectively. **(D)** Principal component analysis of the transcriptome profiles of the three m6A modification patterns. Blue dots represent m6A cluster A, yellow represent m6A cluster B and red represent m6A cluster C. **(E, F)** KEGG enrichment analysis shows the activation status of biological pathways with different m^6^A modification modes. Heat maps are used to visualize these biological processes. Red represents activated pathways and blue represents inhibited pathways. Take rectal cancer GSE87211 cohort as sample annotation. **(E)** m^6^Acluster A vs m^6^Acluster B; **(F)** m^6^Acluster A vs m^6^Acluster C.

We are trying to further determine whether the connection between writers, erasers and readers plays a key role in the formation of different m6A modification patterns, and is related to the formation of TME cell infiltration characteristics and the incidence and progression of cancer. Based on these assumptions, we use the R package of Consensus Cluster Plus to classify patients with qualitatively different m6A modification patterns based on the expression of 23 m6A regulatory factors (**Supplementary Figures 2A–L**). In order to explore the interaction between the 23 m6A regulators in RC patients, the connection between the regulators and their prognostic value, we constructed a m6A regulator network (**Figure 3A**). As we expected, not only the expression of m6A regulatory factors of the same functional category showed a significant correlation, but also a significant correlation among writers, erasers and readers (**Figure 3A**). Among these m6A regulatory factors, the m6A binding protein IGFBP1 has attracted our attention because of its significant correlation with prognosis and immune infiltration (30). Our KaplanMeier survival analysis (p=0.041) showed that patients in the IGFBP1 high expression group had a good prognosis, and we also determined that IGFBP1 was significantly related to the prognosis (**Figure 3B**). We also analyzed the unsupervised aggregation of 23 m6A regulatory factors in the GSE87211 rectal cancer cohort and the TCGA cohort. The survival status, tumor stage, gender, age, project, and m6Acluster were used as patient annotations (**Figure 3C**). The results showed that most elderly male patients with advanced survival in ZC3H13, LRPPRC, HNRNPA2B1 and RBMX were highly expressed in m6Aclusters-A, and METTL14, YTHDC1, YTHDC2, YTHDF3 and in ALKBH5, most young male patients with late-stage survival are highly expressed in m6Aclusters-B, while most young male patients with late-stage survival in VIRMA, YTHDF1, FMR1, IGFBP1, IGFBP2, IGFBP3, and FTO are highly expressed in m6Aclusters-C (**Figure 3C**). The results show that there is an inseparable connection between m6A regulatory factors and clinical characteristics. In addition, the principal component analysis (PCA) of the transcriptome profiles of the three m6A modification patterns showed that the transcriptomes of different modification patterns are significantly different (**Figure 3D**).

### Characteristics of m6A Modification Mode

**Figure 4:**
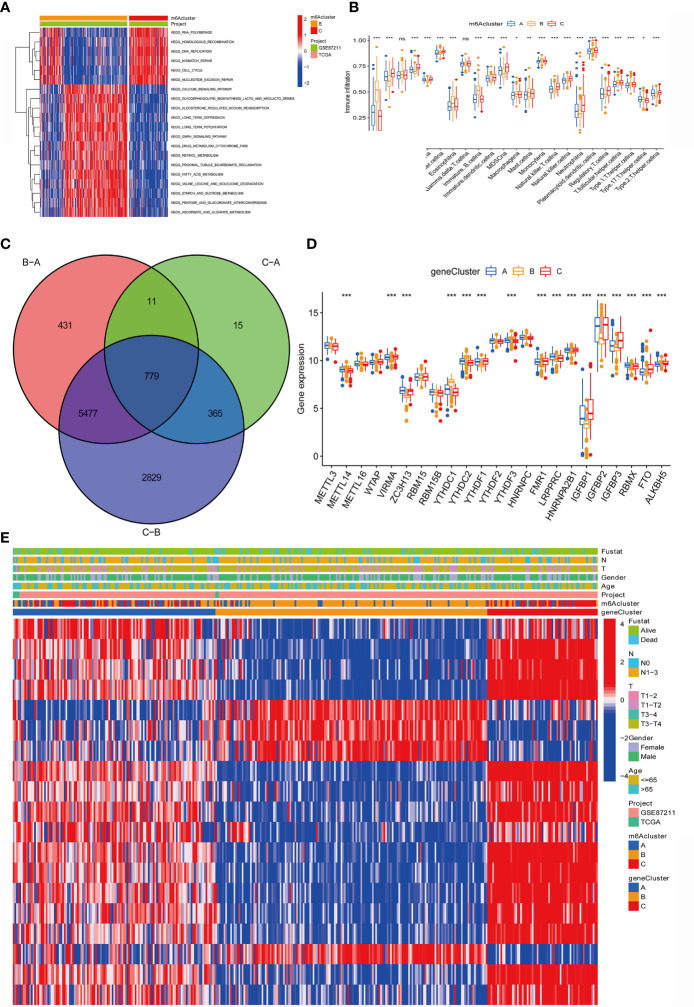
Construction of m^6^A differential gene expression and gene cluster. **(A)**The heat maps show the differences in KEGG enrichment analysis results between the m^6^Acluster B and m^6^Acluster C. **(B)** The abundance of each TME infiltrated cell in the three m6A modification modes. The upper and lower ends of the box represent a quarter of the value range. The line in the box represents the median value, and the dots outside the boxes and their vertical lines represent the outliers. The ordinate shows the immune infiltration while the abscissa represents various types of immune cells. Asterisks indicate statistical P values (*P < 0.05; **P < 0.01; ***P < 0.001). "ns" means "no significance". **(C)** 779 m^6^A-related differentially expressed genes (DEGs) are displayed in the intersection of Venn diagram among the three m^6^A clusters. The red circle represents the DEGs between patients of m6A cluster B and cluster A. The green circle represents the DEGs between patients of m6A cluster C and cluster A. The purple circle represents the DEGs between patients of m6A cluster B and cluster C. **(D)** Expression of 23 m^6^A regulatory factors in three gene clusters. The upper and lower ends of the box represent a quarter of the value range. The line in the box represents the median value, and the black dots represent the outliers. The ordinate represents the level of gene expression in a gene cluster while the abscissa represents the 23 m6A regulatory factors. Asterisks indicate statistical P values (*P < 0.05; **P < 0.01; ***P < 0.001). One-way ANOVA was used to test the statistical differences among the three gene clusters. **(E)** Unsupervised aggregation in the GSE87211 rectal cancer cohort, using survival status, tumor stage, gender, age, project, m^6^Acluster, and gene cluster as patient annotations. Red represents high expression of regulatory factors, and blue represents low expression.

In order to determine the biomolecule changes under the three different m6A modification modes, we performed GSVA enrichment analysis on the gene set, as shown in **Figures 3E, F**, **4A**. The results showed that m6Acluster-A was significantly enriched in Nucleotide excision repair, RNA polymerase, degradation, Cell cycle, Base excision repair, One carbon pool by folate, DNA replication, and Homologous recombination (**Figures 3E, F**). m6Acluster-B is used in FC epsilon Ri, GNRH, calcium and neurotrophin signaling pathway, aldosterone regulated sodium reabsorption, long term potentiation and depression, proximal tubule bicarbonate reclamation, starch and sucrose, fatty acid, retinol, ascorbate and aldarate metabolism, vascular muscle contraction, and endocytosis and other processes are significantly enriched (**Figures 3E**, **4A**). However, m6Acluster-C is significantly enriched in Toll like and Nod receptor signaling pathway, mismatch repair, DNA replication, cell cycle, nuclear excision repair, RNA polymerase and homologous recombination (**Figures 3F**, **4A**).

In addition, we also analyzed the infiltration of TME cells (**Figure 4B**). We noticed that m6Aclusters-C has abundant innate immune cell infiltration, including natural killer cells, MDSC, regulatory T cells, neutrophils, and type 2 T cells. Helper cells, Immature dendritic cell, and CD56 bright and dim natural killer cell. We also noticed significant increases in Activated B cell, Eosinophils, Immature B cell, Macrophages, Mast cells and Monocytes in m6Aclusters-B. However, it is surprising that m6Aclusters-A is significantly higher than m6Aclusters-B and m6Aclusters-C. The degree of infiltration in immune cells is low.

Furthermore, in the above results, it is found that there is a strong positive correlation between IGFBP1 and YTHDF1 (**Figure 3A**). Previous studies have shown that the m6A regulatory factor YTHDF1 mediates the activation of dendritic cells (DC) and the mechanism of CD8+ T cell antigen cross-priming by enhancing the translation of cathepsin (lysosomal protease that degrades antigens in the phagosome) mRNA encoding. Interestingly, this study noticed that IGFBP1 was significantly increased in immune infiltration in Activated B cell, Eosinophils, Immature B cell, Macrophages, Mast cells and Monocytes (**Figure 4B**).

GSVA analysis showed that IGF2BP1 is involved in FC epsilon ri, GNRH, calcium and neurotrophin signaling pathway, aldosterone regulated sodium reabsorption, long term potentiation and depression, proximal tubule bicarbonate reclamation, starch and sucrose, fatty acid, retinol, ascorbate and aldarate metabolism, vascular muscle Significantly enriched during contraction, and endocytosis (**Figures 3E, F**, **4A**). In summary, we speculate that IGFBP1 may cooperate with YTHDF1 to mediate methylation modification, thereby inhibiting the activation of DCs and cytotoxic T lymphocytes, hindering the anti-tumor immune response in tumors.

### m6A Phenotype-Related DEGs in Rectal Cancer

The above study classified m6A-regulated gene expression consensus clustering algorithm into three m6A-modified phenotypes, but the potential genetic changes and expression perturbations in these phenotypes are still unclear. Therefore, it is necessary to further analyze the possible m6A-related transcriptional expression changes of the three m6A modification patterns in RC. We used the empirical Bayes method to determine the overlapping differentially expressed genes (DEGs) among the three m6A modification patterns. Expressed as a Venn diagram, the inclusion of 779 DEGs represents the key distinguishing index of the three m6A modification modes (**Figure 4C**). And we analyzed the expression of 23 m6A regulatory factors in 3 gene clusters (**Figure 4D**). The results show that compared with gene Cluster-B, the expression of ZC3H13, LRPPRC, HNRNPA2B1 and RBMX in gene Cluster C were significantly higher. YTHDC1, YTHDC2, YTHDF3 and ALKBH5 in gene Cluster-B was higher than the other two gene clusters. Compared with gene Cluster-A and gene Cluster-B, VIRMA, YTHDF1, FMR1, IGFBP1, IGFBP2, IGFBP3 and FTO were significantly increased in gene Cluster-C.

**Figure 5:**
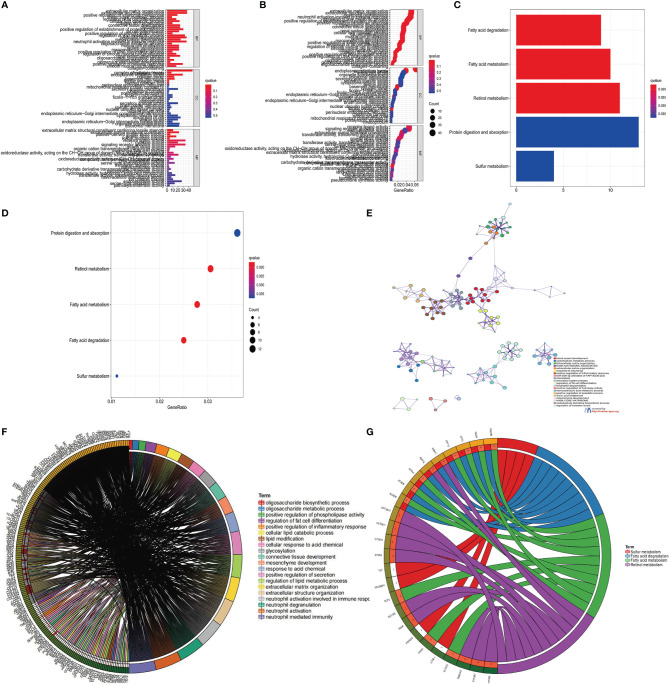
KEGG and GO enrichment analysis of m6A related genes. **(A, B)** GO enrichment analysis for functional annotation of m^6^A-related genes. The color depth of the histogram represents the number of enriched genes. **(C, D)** KEGG enrichment analysis for the functional annotation of m^6^A-related genes. The color depth of the histogram represents the number of enriched genes. **(E)** The interaction of pathways of 23 m6A regulatory factors. The circles of different colors represent different types of pathways and the lines linking them represent their interaction. **(F)** Circle plot showing the enrichment analysis of various pathways. **(G)** Circle plot showing the enrichment analysis of different metabolic pathways.

In addition, we also analyzed three clinicopathological characteristics, and we found that male patients with advanced clinical stages (N1-3, T3-T4) less than or equal to 65 years old are mainly concentrated in gene Cluster-C. Male patients over 65 years old are mainly concentrated in gene Cluster-B (**Figure 4E**). Next, we will conduct follow-up research and analysis on these characteristic genes, GO enrichment analysis shows: in extracellular matrix organization, extracellular structure organization, positive regulation of inflammatory response, collagen-containing extracellular matrix, complex of collagen trimers, extracellular matrix structural constituent conferring tensile strength, extracellular matrix structural constituent, growth factor binding and RAGE. The biological process of receptor binding is significantly more common (**Figures 5A, B**). KEGG enrichment analysis showed that: Fatty acid degradation and metabolism, Retinol and Sulfur metabolism and Protein digestion and absorption biological processes are significantly enriched (**Figures 5C, D**). Next, we use the R package to further analyze the pathway enrichment of 23 m6A regulatory factors, and visualize it with ggpolt, and get similar results (**Figure 5E**). The enrichment analysis of various cellular pathways was shown in circle plot (**Figure 5F**) and another circle plot indicated the enrichment analysis of metabolism (**Figure 5G**). The above further confirmed that the overlapping dimer has m6A modification and immune characteristics, similar to the characteristics of m6A-related genes. We noticed that there are significant differences in m6A regulatory gene expression among the three m6A gene signature subgroups, which is consistent with the expected results of m6A methylation modification patterns.

### Construction and Application of m6Ascore

**Figure 6:**
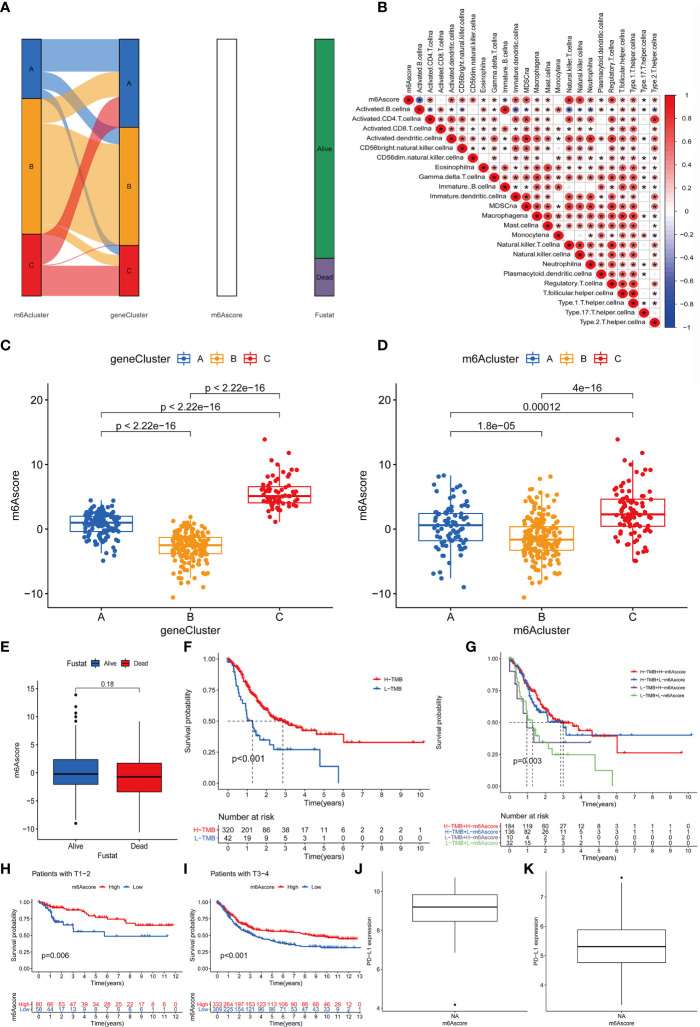
Construction of m6A score and analysis of related modification patterns and clinical treatment effect of m6A score. **(A)** The alluvial map shows the changes of m^6^Aclusters, gene clusters, m^6^Ascore and survival status. **(B)** Using Spearman analysis, the correlation between m6score and known gene characteristics in the GSE87211 cohort. Blue is a negative correlation, and red is a positive correlation P values (*P <0.05; **P <0.01; ***P <0.001). **(C)** The difference of gene clusters among the three gene clusters in the GSE87211 cohort. The ordinate represents the m6A score while the abscissa shows the gene cluster A–C. Kruskal Wallis test was used to compare the statistical differences between the three gene clusters (P<0.001). **(D)** The difference of m^6^Acluster among the three gene clusters in the GSE87211 cohort. The ordinate indicates the m6A score while the abscissa shows the three m6A cluster A–C. Kruskal Wallis test was used to compare the statistical differences between the three gene clusters (P<0.001). **(E)** The difference of m6Ascore between different survival states in the GSE87211 cohort. **(F)** Kaplan-Meier curve of TMB high and low patients in the TCGA cohort, Log-rank test, P<0.001. The numbers of patients with high and low TMB were shown in the bottom. **(G)** Kaplan-Meier curve of patients with high and low TMB-m6Ascore in the TCGA cohort, Log-rank test, P=0.003. **(H)** Kaplan-Meier curve of patients with high and low m6Ascore in patients with T1-2 stage of rectal cancer. Log-rank test, P = 0.006. **(I)** Kaplan-Meier curve of patients with high and low m6Ascore in patients with rectal cancer T3-4 stage. Log-rank test, P<0.001. **(J, K)** PD-L1 expression difference in m6Ascore group.

The above results indicate that m6A methylation modification plays a crucial regulatory role in the TME landscape. However, these results are based on patient populations and are not suitable for evaluating the m6A methylation modification pattern of a single patient. In addition, considering that the research process may be interfered by individual heterogeneity and complexity, we have constructed a set of the scoring scheme for quantifying the m6A modification pattern of individual patients with rectal cancer was named m6Ascore. The alluvial map is used to illustrate the workflow of m6Ascore construction (**Figure 6A**). In order to better illustrate the characteristics of m6A signatures, we also tested the correlation between known signatures and m6Ascore (**Figure 6B**). These results indicate that gene Cluster-C has the highest m6Ascore, followed by gene Cluster-A and gene Cluster-B (**Figure 6C**). It is worth noting that m6Acluster-A, m6Acluster-B and m6Acluster-C obtained similar results (**Figure 6D**).

In addition, we tried to explore the significance of m6Ascore in clinical work. We first analyzed the relationship between m6Ascore and patient survival status, and found that m6Ascore had no significant difference between survival and death groups (p=0.18) (**Figure 6E**). Previous studies have shown that there is a close relationship between tumor genome cell mutations and immunotherapy response. Therefore, we explored the distribution of tumor mutation burden in different m6Sig score groups and found that regardless of the status of m6A, the H-TMB group had a better prognosis (**Figure 6F**). Further analysis showed that in the H-TMB group, there was no significant difference in the prognosis of patients in the H-m6Ascore and L-m6Ascore, while in the L-TMB group, the H-m6Ascore group had a better prognosis than the L-m6Ascore group(p=0.003) (**Figure 6G**). This is consistent with the results of previous studies, that is, high mutation load is positive correlation with in immune evasion and tumor cell proliferation (31). Finally, we further evaluated the relationship of m6Ascore in tumor staging of T1-2 and T3-4 patients, and the results showed that the prognosis of patients with stage T1-2(p=0.006) and stage T3-4(p<0.001) is better under high m6Ascore (**Figures 6H, I**). The above results strongly suggest that m6Ascore can represent the change pattern of m6A and predict the prognosis of RC patients. These data allow us to more fully describe the impact of m6Ascore classification on genomic variation and reveal the possible complex interactions between individual cell mutations and m6A modifications.

### The Role of m6Ascore in Predicting Immunotherapeutic Benefits

Next, consider that PD-L1 is a mature biomarker predicting response to anti-PD-1/L1 treatment, the emergence of immunotherapy represented by PD-L1 and PD-1 block is undoubtedly a major breakthrough in cancer treatment. We further compared the relationship between different m6Ascore and PD-L1 expression level and found that the higher the m6Ascore, the higher the PD-L1 expression level (**Figures 6J, K**). In summary, our research results show that there is a close relationship between m6Ascore and immune response, which can be used in clinical work to evaluate immune response and further predict the prognosis of patients.

## Discussion

Many recent studies have shown that m6A modification interacts with m6A regulatory factors and plays a vital role in immune, inflammation and cancer treatment (22, 32, 33). So far, most published studies have focused on unilateral studies of TME cell types and regulatory factors, but the overall characterization of TME infiltration mediated by the combined action of multiple m6A regulatory factors is far from enough. Therefore, it is of great significance to further explore the role of different m6A modification modes in TME cell infiltration, which will help us to improve our understanding of the anti-tumor immune response of TME and open up a new path for the immunotherapy of malignant tumors.

In this study, we summarized three different m6A methylation modification patterns, which have their own unique characteristics of TME cell infiltration. m6Aclusters-C has a rich infiltration of innate immune cells, including natural killer cells, MDSC, regulatory T cells, neutrophils, type 2 T helper cells, Immature dendritic cells, and CD56 bright and dim natural killer cells. We also noticed significant increases in Activated B cell, Eosinophilna, Immature B cell, Macrophagena, Mast cellna and Monocytena in m6Aclusters-B. However, it is surprising that m6Aclusters-A is significantly higher than m6Aclusters-B and m6Aclusters-C. The degree of infiltration in immune cells is low. Previous studies have shown that the tumor microenvironment plays an essential role in tumor progression and the effect of immunotherapy. CD8+ T lymphocytes are triggered by specific dendritic cells in the tertiary lymphatic structure (TLS) located in the tumor. Other cell types (such as CD4+ T cells) may also contribute to immune surveillance, thereby enhancing the anti-cancer immune response ability (34). The baseline levels of tumor-infiltrating CD8+ T cells, CD4+ T cells, and NK cells are related to the likelihood of an immune response (35–37). The intratumoral immune landscape, including memory cells, cytotoxic cells [CD8+ T cells, natural killer (NK) cells and NK T (NKT) cells] and immunosuppressive cells [Tregs and myeloid-derived suppressor cells (MDSCs)], quantitative Immune core prognostic markers predict survival more accurately than standard TNM staging (25).

In addition, we also analyzed the enrichment of m6A modification patterns in immune-related biological pathways. m6Acluster-A is significantly enriched in Nucleotide excision repair, cell cycle regulation and Homologous recombination. The feature of m6Acluster-B is that it is significantly enriched in the process of calcium and neurotrophin signaling pathway, aldosterone regulated sodium reabsorption and endocytosis. And m6Acluster-C is significantly enriched in Toll like and Nod receptor signaling pathway, mismatch repair, and DNA replication. Combination studies have shown that mismatch repair defects and Toll like signaling pathway both lead to higher tumor mutation burden and immune response (38–41). The TME is also related to the response to immune checkpoint block (ICB) therapy. Our research is consistent with the above results. Considering the individual heterogeneity of m6A modification, it is necessary to quantify the m6A modification pattern of a single tumor. In view of that, we have established a scoring system to evaluate the m6A modification patterns of individual rectal cancer patients-m6A gene signature, in order to better guide the treatment strategies of individual rectal cancer patients. The results showed that m6Ascore of m6Acluster-C was the highest, followed by m6Acluster-A and m6Acluster-B. In addition, gene clusters constructed from differentially expressed genes (DEGs) identified from different m6A modification patterns have obtained similar results to m6A modification clusters. We also analyzed the prognosis of the m6Ascore high and low groups and found that the high m6Ascore group has a clear survival advantage. This further shows that m6Ascore is a promising tool, and m6Ascore is a prognostic biomarker for RC.

Tumor mutation burden correlates with immunotherapy response (42), in addition, it has been confirmed that programmed death receptor ligand 1 (PD-L1) interacts with the tumor microenvironment to mediate tumor immune escape. PD-L1 inhibitors are a hot spot in tumor immunotherapy in recent years. They can restore the activity of T cells and enhance the body’s immune response (43). Here we found that m6Ascore, the higher the expression of PD-L1, which means that m6Ascore can guide immunotherapy. It is worth noting that we also found that m6Ascore can be used to assess the clinicopathological characteristics of patients, including survival status, gender, age, and tumor stage. This research provides a new perspective for the development of new drugs and immunotherapy, and brings hope to the precise treatment of clinical malignant tumors, the identification of different tumor immunophenotypes, and the improvement of individualized tumor immunotherapy.

It must be admitted that our analysis also has potential limitations. First of all, our study is retrospective. Therefore, a prospective cohort of RC patients receiving immunotherapy is needed to verify our results. Secondly, the newly discovered regulatory factors need to be incorporated into the model in future research to optimize the accuracy of the m6A modification pattern. In addition, it is necessary to explore a suitable rectal cancer data set to verify the effect of m6Ascores from various clinical aspects, so as to further strengthen our conclusions. In summary, in this study, for the first time, we constructed 23 RNA methylation regulators with rectal cancer as the research object, analyzed the m6A modification patterns of 369 rectal cancer samples, and systematically compared these modification patterns with the characteristics of TME cell infiltration are related to clinicopathological characteristics. This research helps to enhance our understanding of the characteristics of TME infiltration and provides new insights for more effective individualized immunotherapy strategies.

## Data Availability Statement

The datasets presented in this study can be found in online repositories. The names of the repository/repositories and accession number(s) can be found in the article/**Supplementary Material**.

## Author Contributions

KL, JH,YH and SY have contributed equally to this work and share first authorship. KL and JH: Design research direction, Writing papers; YH and SY: Data analysis, Drawing figures; QY, FS, CW, YC, WZ, XZ and HL: Searching for references, Helping to write papers; XW: Review and revise the papers, Guidance article writing. All authors contributed to the article and approved the submitted version.

## Funding

This study was funded by the Natural Science Foundation of China. (No. 81860034 to XW).

## Conflict of Interest

The authors declare that the research was conducted in the absence of any commercial or financial relationships that could be construed as a potential conflict of interest.

## Publisher’s Note

All claims expressed in this article are solely those of the authors and do not necessarily represent those of their affiliated organizations, or those of the publisher, the editors and the reviewers. Any product that may be evaluated in this article, or claim that may be made by its manufacturer, is not guaranteed or endorsed by the publisher.

## Supplementary Material

Supplementary Figure 1 The expression of 23 m^6^A regulatory factors in ZC3H13 Wild. **(A–W) **The upper and lower ends of the box represent a quarter of the value range. The line in the box represents the median value, and the black dots represent the outliers.

Supplementary Figure 2 Unsupervised cluster analysis of m^6^A regulatory factors.** (A)** Consensus matrix legend. **(B–I)** Consensus matrices of the TCGA and GSE87211 cohort for k = 2-9. **(J)** The consensus graph of CDF value K=2-9. **(K)** CDF curve with CDF value K=2-9. **(L)** CDF value K=2-9 in the tracking plot of the samples.

## Abbreviations

m6A, N6-methyladenosine; RC, rectal cancer; TME, tumor microenvironment; PCA, principal component analysis; CRC, colorectal cancer; TCGA, The Cancer Genome Atlas; GEO, Gene-Expression Omnibus; GSVA, Gene set variation analysis; GO, Gene Ontology; KEGG, Kyoto encyclopedia of genes and genomes; ssGSEA, single sample gene set enrichment analysis; DEGs,differentially expressed genes; DCs, Dendritic cells; TLS, tertiary lymphatic structure; NK, natural killer; ICB, Immunological checkpoint blockade; PD-L1, programmed death receptor ligand 1; CNV, copy variation; MDSCs, myeloid-derived suppressor cells.
