# Supplementary figures and images for: m6A Regulator-Based Methylation Modification Patterns Characterized by Distinct Tumor Microenvironment Immune Profiles in Rectal Cancer
Source: Front Oncol. 2022 Jul 6;12:879405. doi: 10.3389/fonc.2022.879405 (PMC9299953; doi:10.3389/fonc.2022.879405)

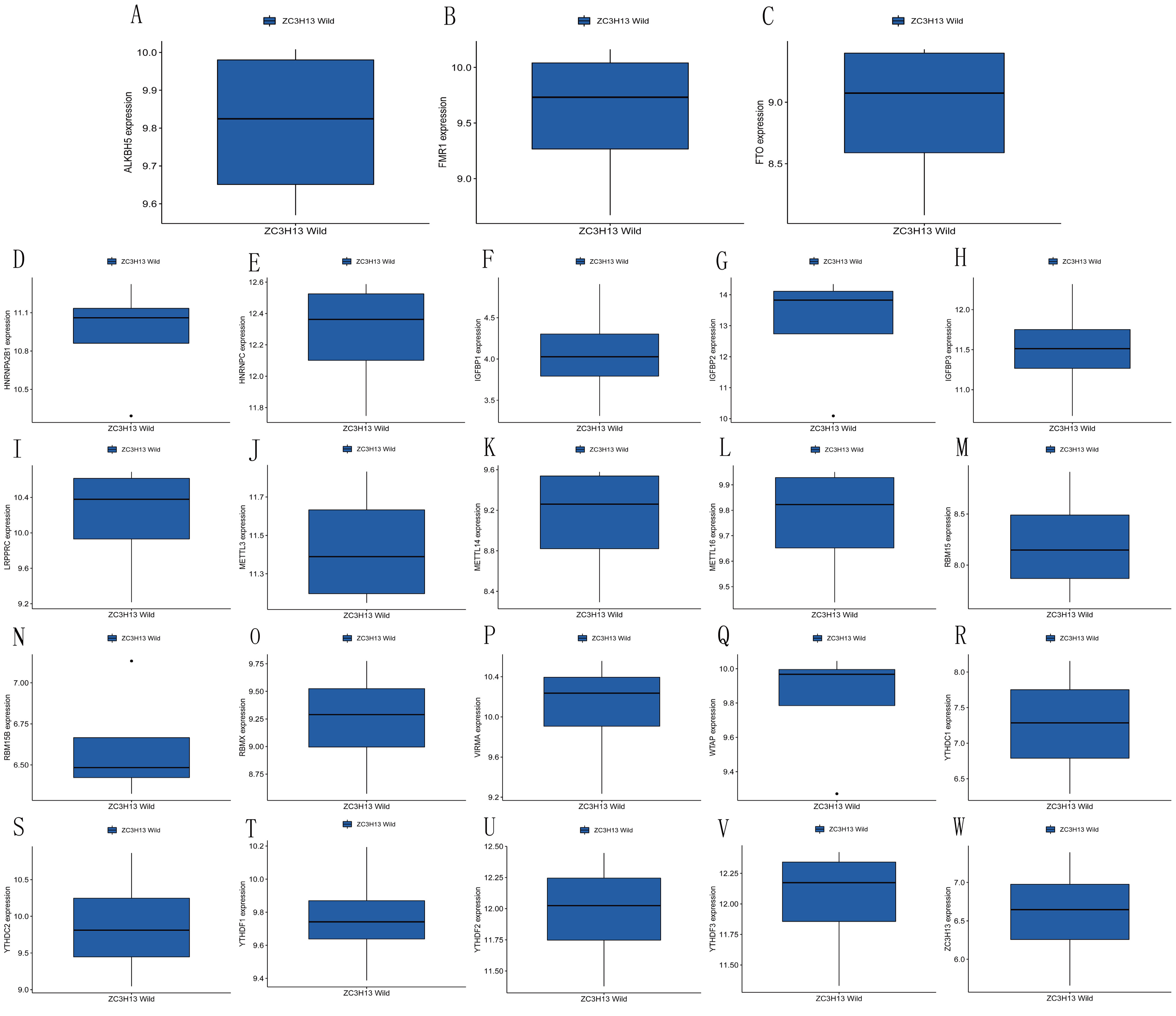

Supplement: Supplementary Figure 1 — The expression of 23 m6A regulatory factors in ZC3H13 Wild. (A–W) The upper and lower ends of the box represent a quarter of the value range. The line in the box represents the median value, and the black dots represent the outliers. [file Image_1.jpeg]

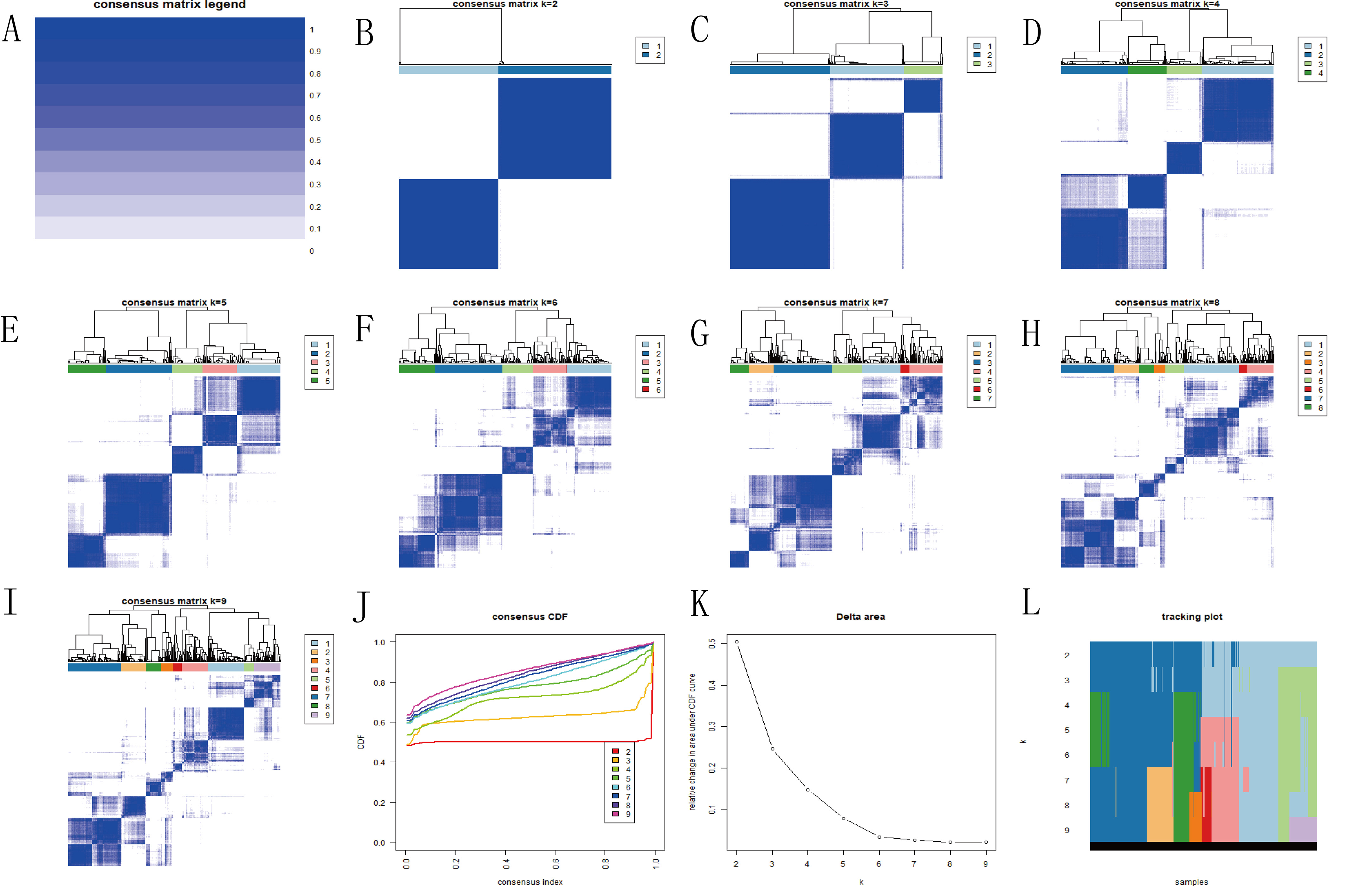

Supplement: Supplementary Figure 2 — Unsupervised cluster analysis of m6A regulatory factors. (A) Consensus matrix legend. (B–I) Consensus matrices of the TCGA and GSE87211 cohort for k = 2-9. (J) The consensus graph of CDF value K=2-9. (K) CDF curve with CDF value K=2-9. (L) CDF value K=2-9 in the tracking plot of the samples. [file Image_2.jpeg]
